# Effect of Aspect Ratio on the Deformation Behavior of Dislocation-Free Ni_3_Al Nanocubes

**DOI:** 10.3390/nano10112230

**Published:** 2020-11-10

**Authors:** Peng Li, Xinguang Wang, Yizhou Zhou, Janine Pfetzing-Micklich, Christoph Somsen, Gunther Eggeler

**Affiliations:** 1 Clean Nano Energy Center, State Key Laboratory of Metastable Materials Science and Technology, Yanshan University, Qinhuangdao 066004, China; 2 Institut für Werkstoffe, Ruhr-Universität Bochum, 44780 Bochum, Germany; janine.pfetzing@rub.de (J.P.-M.); christoph.somsen@ruhr-uni-bochum.de (C.S.); gunther.eggeler@ruhr-uni-bochum.de (G.E.); 3 Shi-Changxu Innovation Center for Advanced Materials, Institute of Metal Research, Chinese Academy of Sciences, Shenyang 110016, China; yzzhou@imr.ac.cn

**Keywords:** Ni_3_Al nanocubes, Ni-base superalloy single crystals, aspect ratio, in situ compression, dislocation free

## Abstract

This study concentrates on several factors which govern the nanoscale plasticity of in situ compressed dislocation-free Ni_3_Al nanocubes: cube size, aspect ratio and the presence of grooves. The yield strength of dislocation-free Ni_3_Al nanocubes exhibits an apparent size dependence. The size dependence is strong when cubes are smaller than 300 nm. Compared with the strength of bulk Ni_3_Al single crystals, the strength of nanocubes is two orders of magnitude higher, which clearly demonstrates that there is a size effect. Nanocube plasticity strongly depends on the alignment and the shape of the cubes. Deformed aligned nanocubes either display only a few localized deformation events (slip lines) or were homogenously compressed into flats due to multiple slip dislocation-mediated plasticity. For an aligned cube, crack initiation at the intersection of a slip line with a groove in the cube surface was observed. In case of a double cube, crack initiation occurs at surface irregularities, while subsequent crack propagation occurs along one or more slip planes.

## 1. Introduction

Ni-based superalloy single crystals (SXs) have been widely used in turbine blades for aero engines and gas turbines for energy production because of their excellent high-temperature strength. The typical microstructure of SX consists of L1_2_-ordered γ’ (Ni3(Al, Ti)) cubes coherently embedded in a γ matrix with a face-centered cubic crystal structure [1]. The mechanical properties of these materials depend on the volume fraction, the spatial arrangement and the size and morphology of the γ’ precipitates. As the microstructure plays an important role in the mechanical properties of the alloys, it is therefore worth studying the effects of the microstructure more closely [2].

**Figure 1:**
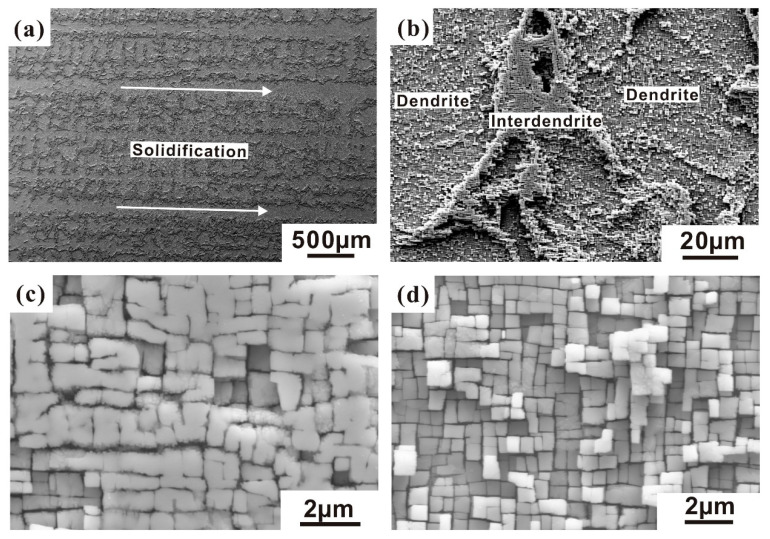
Secondary electron SEM micrographs of cast microstructures in single crystals (SX). (**a**,**b**) Dendrites and interdendritic regions; (**c**) microstructure in the interdendritic region; (**d**) microstructure in the dendrites.

Firstly, the cast microstructure of the alloys is governed by the growth of dendrites (D) and the subsequent solidification of interdendritic (ID) regions. Chemical and microstructural differences between these two regions result in large-scale heterogeneity, which is an inherent feature of SX microstructures. As shown in Figure 1a, the typical average spacing between the centers of individual dendrites is of the order 400 μm [3]. Figure 1b exhibits a microstructure which was taken parallel to the [001] solidification direction using secondary electron contrast in the scanning electron microscope (SEM). It is well known that heavy elements like Re and W segregate to the dendrite core while light elements like Al and Ti have a tendency to partition to the interdendritic regions [4,5,6].

Comparing Figure 1c,d, one finds that the size and morphology of the γ’ precipitates show distinct differences. In the interdendritic region, it is found that the γ’ precipitates are larger and often in close contact because of higher Al content. Therefore, for technical applications, multiple step heat treatments are required to homogenize SX alloys with respect to the chemical differences between D and ID regions after solidification [3]. During the precipitation heat treatment of SXs, a fine γ/γ’-microstructure forms, where the volume fraction of γ’ precipitates is typically 70%, their edge length is about 0.5 μm and the γ/γ’ interfaces are parallel to {001} planes [7,8].

Razumovskii et al. [9] summarized the relations between yield strength and particle size, which can be presented as [10]:

(1) $$\tau =\alpha \cdot \gamma_{APB}^{3/2}\cdot f^{1/2}\cdot r^{1/2}$$

where $\alpha =0.28\cdot G^{-1/2}\cdot b^{-2}$, $\gamma_{APB}$ denotes the antiphase boundary energy, *f* is the volume fraction and *r* is the average size of particles. The parabolic *r*^1/2^ dependence suggests that larger precipitates are associated with higher critical stresses. This holds until another mechanism, the Orowan mechanism, operates. The critical stress for Orowan bypassing by single dislocations falls off as 1/*r*. Therefore, there is an optimum γ’ particle size, which provides maximum strength.

After heat treatments, the γ’ precipitates mainly consist of cube-shaped nanoparticles, but one often finds deviation from the perfect cubic shape. It is quite common to find elongated particles, smaller and larger plates, doubles of short plates and so on. This can be accounted for by measuring the aspect ratios of the particles (height to width ratio). To explore the possibility of nano-object forging and to try to achieve close-to-theoretical strengths, Rösler et al. [11] and Maaβ et al. [12] successively carried out in situ compression tests of free-standing Ni_3_Al nanocubes. Maaβ et al. [12] found variation in the elastic response to loading, which they attributed to non-ideal alignment of the indenter probe and various shape imperfections. In fact, most γ’ particles in the superalloy represent imperfect cubes.

In the present work, we investigate the effect of nanocube size and H/W ratio on the in-situ compressive behavior of dislocation-free Ni_3_Al particles (“H”: height of nanocubes; “W”: width of nanocubes, which is simply represented by an average diameter). We determine the best match between nanocube size and H/D ratio, in order to produce higher-strength nanoscale metallic components. Special emphasis is placed on the deformation and fracture mechanisms of dislocation-free Ni_3_Al nanoparticles.

## 2. Material and Experiments

**Figure 2:**
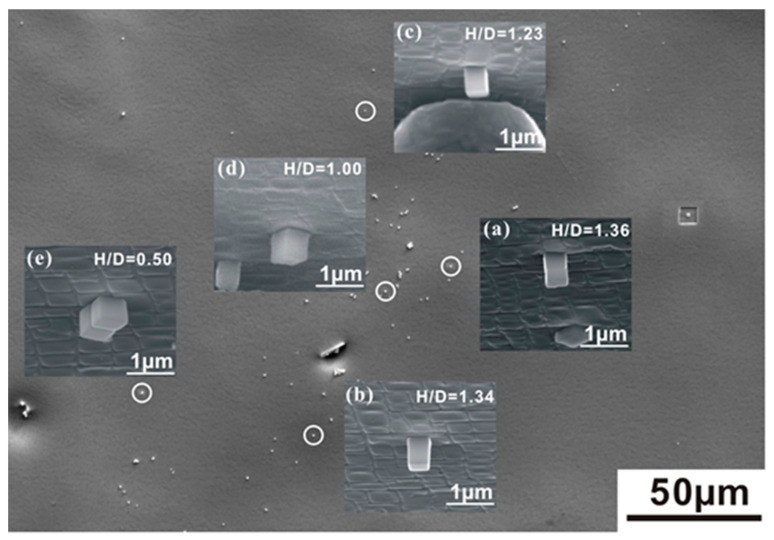
Cube-shaped nanoparticles with different aspect ratios in the size range of 300–500 nm which were isolated from the SX alloy ERBO-1 by selective phase dissolution. (**a**) height/weight (H/W) = 1.36; (**b**) H/W = 1.34; (**c**) H/W = 1.23; (**d**) H/W = 1.00; (**e**) H/W = 0.50.

Discs with 0.5 mm thickness were cut from a SX of type ERBO/1C. Details concerning the alloy composition and the heat treatment of the material are given in the literature [3]. Its microstructure consists of Ni_3_Al-type coherent precipitates. These precipitates were isolated from the alloy by an electrochemical polishing procedure (aqueous electrolyte: HClO_4_:C_3_H_8_O_3_:methanol = 1:2:7; voltage: 16 V), which selectively dissolves the matrix phase and leaves the precipitates behind [13]. As a result, one finds a few dislocation-free nanoparticles dispersed on the SX surface (Figure 2).

We selected five nanoparticles with different H/W ratios for micro-compression testing. These are referred to as the aligned cube (H/W = 1.36), the long cuboid (H/W = 1.34), the short cuboid (H/W = 1.23), the perfect cube (H/W = 1.00) and the double cube (H/W = 0.50). Note that the aligned cube is composed of two perfect cubes one on top of the other while the double cube consists of two perfect cubes side by side (see Figure 2a,e, respectively). The other three selected compression specimens consist of isolated single Ni_3_Al particles. The in situ compression experiments were performed in a SEM of type Quanta 650 FEG (Thermo Fisher Scientific., Hillsboro, OR, USA). For every size/geometry, three to five similar cubes were tested. This yields SEM images of good quality which can be recorded during testing. High-quality SEM pictures were taken from a SEM of type Leo 1530 VP (Carl Zeiss AG, Obekochen, BW, Germany) at the end of each test. A camera allowed the monitoring of all experiments and the recording of videos. The specimen was mounted on a specimen holder at an angle of 70°. Loads were applied using a micromechanical system of type UNAT-SEM2 from ASMEC (ASMEC., Ulm, Germany). The radius R of the circular flat indenter tip was 2.5 μm and loading was performed at a constant displacement rate of 2 nm s^−1^. Details of the micromechanical test procedure used in the present work are described in Reference [14].

A FEI Quanta 200 3D dual-beam focused ion beam (FIB) instrument was used for the preparation of thin TEM foils. TEM analysis was carried out using a FEI Tecnai Supertwin F20 (Thermo Fisher Scientific, Hillsboro, OR, USA) equipped with a field emission gun and operating at 200 kV. TEM work was performed by exploiting bright field (BF) and high-angle annular dark field (HAADF) image contrasts.

## 3. Results and Discussion

### 3.1. Effect of Cubic Size on the Yield Strength

**Figure 3:**
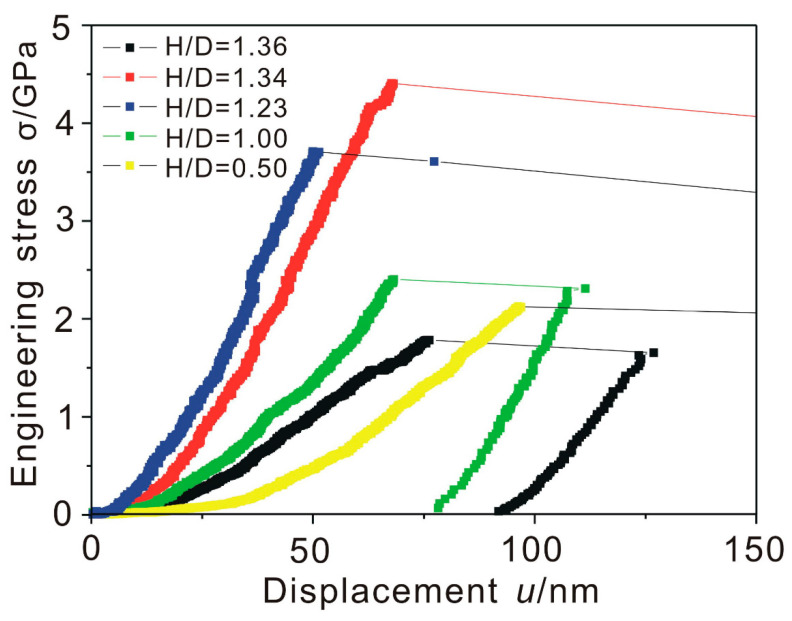
Stress displacement curves for as-extracted Ni_3_Al nanocubes with different aspect ratios.

Figure 3 shows the stress displacement data for the dislocation-free Ni_3_Al nanocubes subjected to in-situ compression along the <001> direction. Compression of dislocation-free particles represents a deformation mode, where the strain in the particle is free of any gradients. As the particles are cube-shaped, the aspect ratio is always close to 1. The micropillar specimens investigated by Bei et al. [15] had higher aspect ratios. They reported that the yield strength of Mo alloy pillars is no longer size-dependent when their size decreases to 1 μm or less. However, the dislocation-free nanocubes display a clear size effect. These nanocubes first deform elastically, followed by plastic yielding, which shows significant scatter. This finding is consistent with the experimental results reported by Maaβ et al. [12]. The transition from the elastic to the plastic regime is associated with activation of the first slip system, which can be observed in the video of in-situ compression. Yield events manifest themselves as long, almost horizontal lines, indicating a large increase in displacement with a small decrease in stress level. Measured axial yield stresses vary between close to 2 to above 4 GPa. Maaβ et al. [12] compared the yield strengths of dislocation-free and dislocated Ni_3_Al nanocubes and concluded that the data obtained from as-extracted nanocubes exhibit a weaker size-dependence than FIB micromachined nanocubes.

**Figure 4:**
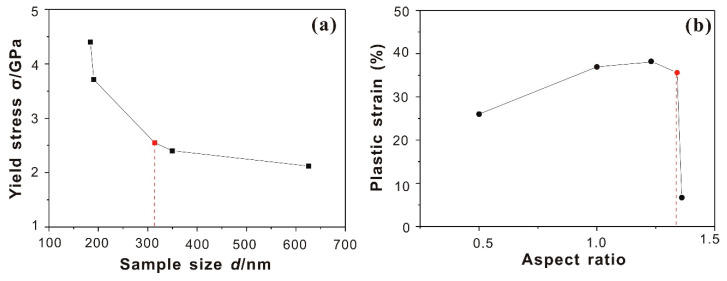
(**a**) Yield stress as a function of cube size, clearly revealing a size effect for dislocation-free Ni_3_Al nanocubes. (**b**) Plastic strain as a function of aspect ratio exploring the effect of aspect ratio on nanocube plasticity.

As shown in Figure 4a, the yield strength of dislocation-free face centered cubic (fcc) nanocubes exhibits a clear size dependence. Due to the non-ideal alignment, the sample with H/W = 1.0 may provide potential sites for dislocation nucleation and multiplication at global stress which are significantly lower than those required for homogeneous dislocation nucleation. Our results suggest that there are size- and shape-dependent transition points of yield strength and plastic strain, respectively (see Figure 4a,b). Below a sample size of 310 nm, there is a strong size dependence of yield stress. The amount of the plastic strain burst increases below an aspect ratio of 1.34. Considering that the shear modulus *G* of Ni_3_Al single crystals is 70.3 GPa, 200-nm nanocubes deformed at the critical resolved shear stress of *G*/32, which is almost twice than that of 600-nm nanocubes. The yield strength for dislocation-free super-350-nm Ni_3_Al nanocubes has a relatively weak size dependence, which is consistent with the conclusions of Maaβ et al. [12]. The results obtained in the present work show that when the size of isolated Ni_3_Al nanocubes falls below 300 nm, the size dependence significantly increases. Compared with the strength value of bulk Ni_3_Al single crystals [16], the normalized deformation strengths of nanocubes is one to two orders of magnitude higher, which demonstrates the universal presence of a size effect. It is therefore reasonable to assume that the superalloy composed of cubes which are smaller than 200 nm show better mechanical behaviors.

Wen and Wang et al. [17] performed first principle calculations to determine the ideal uniaxial compressive strength of Ni_3_Al under [100], [110] and [111] compressive loads. According to their results, the ideal compressive strength of [100]-oriented Ni_3_Al is close to 6.53 GPa. According to Wen et al.’s calculation [18], the theoretical shear strength for single crystals of Ni_3_Al may be extracted as below: they amount to 3.90 GPa for the microscopic crystallographic slip system $\{111\}[11\bar{2}]$ and 4.55 GPa for the system $\{111\}[1\bar{1}0]$, which may be considered as the upper limits of the true theoretical shear strength. Compared with the theoretical strength of bulk Ni_3_Al single crystals, the present experimental strength approaches the limits computed for a defect-free material. However, it seems that the ideal shear strength is more relevant for the present experiments. The ideal compression strength can probably only be obtained when slip events can be fully suppressed during deformation.

### 3.2. Effect of Aspect Ratio on Plasticity

Figure 4b summarized the results which address the dependence of plastic strain on the aspect ratio. Unlike the yield strength, the plastic strain mostly depends on the alignment and the shape of cubes. The plastic strain is determined by the height difference of cubes before and after in-situ compression divided by the original height. Kuzmin et al. [19] were the first to show that there specimens with aspect ratios between 2 to 4 are most suitable for proper compression experiments. For aspect ratios below 2, there is a lower probability for the formation of shear bands. Aspect ratios in excess of 4 promote uncontrollable buckling and superimposed bending stresses. Pillar experiments are always prone to the inhomogeneous evolution associated with bending and buckling and make it difficult to create clean axial compression data. Kuzmin et al. [19] thought that the ductility of bulk metallic glasses (BMGs) at different scales depends on the sample geometry. For the Ni_3_Al nanocubes, the range of aspect ratios is usually below 2; their plastic strain likewise depends strongly on the aspect ratio. It can be found from Figure 4b that with an increasing aspect ratio, the plastic strain first increases and subsequently slightly decreases. At H/W ≈ 1.3, the plastic strain amplitude reaches a maximum, which is close to 40%. Due to the non-ideal alignment, the sample with a 1.0 aspect ratio has a lower ductility than expected. Similarly, for the specimen referred to as the aligned cube, the non-ideal aligned compression only produces plastic strains of 6–7%.

**Figure 5:**
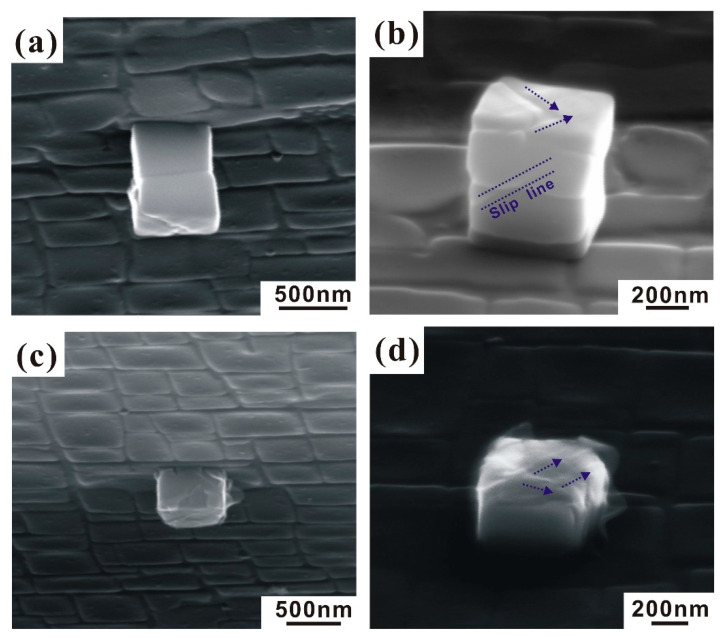
Slip morphologies of as-extracted Ni_3_Al nanocubes after compression. (**a**,**b**) Aligned cube assembly consisting of two particles; (**c**,**d**) long single-particle cuboid.

The difference is closely related to the shape and alignment of Ni_3_Al nanocubes. As indicated in Figure 5a,b, the deformed aligned nanocubes display only a few slip lines and the assembly does not break and almost keeps its shape. One slip line was found which traverses both γ’ particles of the aligned assembly. In addition to the conclusion of Maaβ et al. [12], we find that not only FIB micromachining of Ni_3_Al pillars shows a high scatter of yield stresses. The same is observed in as-extracted nanocubes with different shapes. The aligned cube assembly consists of two γ’ particles and the γ phase between them. Although its aspect ratio is similar to the long cuboid, the deformation process is very different. Figure 5c,d exhibits the slip morphologies of the long single-particle cuboid after deformation. Different oriented slip lines indicate that multiple slip systems were activated. This type of as-extracted nanocubes was homogeneously compressed into flatter shapes by multiple slip dislocation-mediated plasticity, which finally leads to post-yield catastrophic failure.

**Figure 6:**
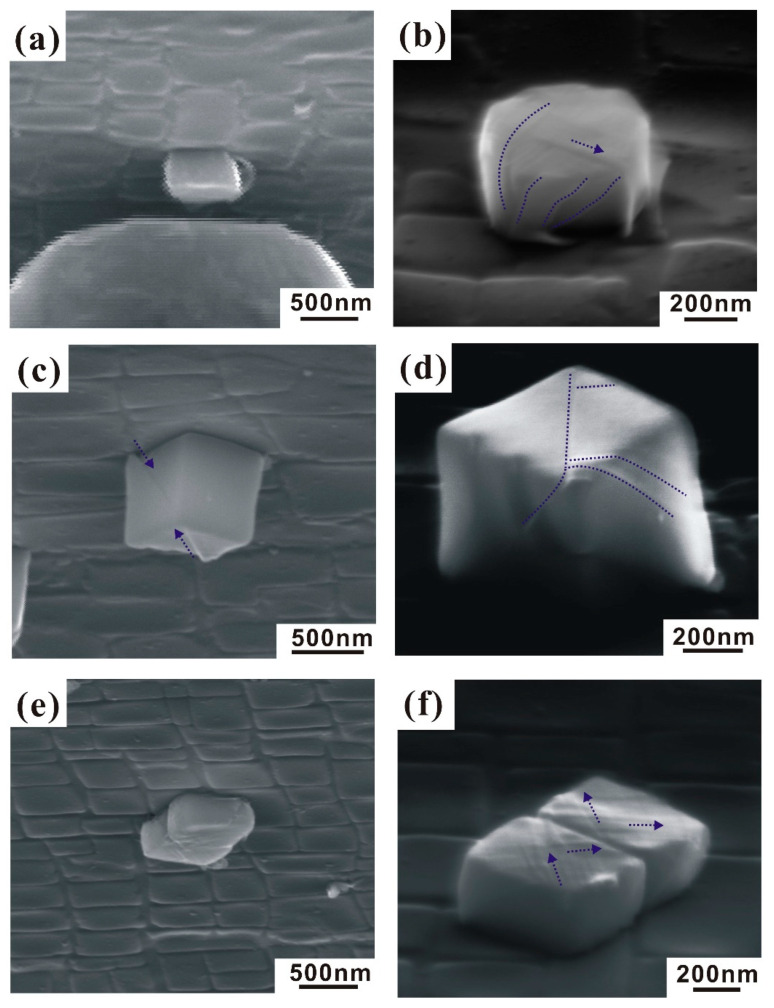
Slip morphologies of as-extracted Ni_3_Al nanocubes after compression. (**a**,**b**) Short cuboid; (**c**,**d**) perfect cube; (**e**,**f**) double cube.

Likewise, numerous slip lines can be observed on the surfaces of the dislocation-free short cuboid (see Figure 6a,b). It should be noted that no resolvable slip lines appear on the dislocation-free Mo-alloy micropillars [15] and Au microspheres [20]. For the as-extracted nanocubes, the relatively low aspect ratio confines a large fraction of slip planes to the region between the punch and the substrate. Multiple nucleation and glide of dislocations along one specific slip plane are so limited that slip proceeds on different slip systems at different locations within the nanocubes, resulting in the apparent homogeneous deformation. On the other hand, the geometrical constraints would influence the cross-slip of dislocations and further suppress the formation of dislocation pile-ups. Thereby, at the beginning of plastic deformation, the nanocube’s symmetry is preserved because of the equal nucleation and propagation probabilities. However, when the aspect ratio is higher, more dislocations can glide away from the crystal’s interior and the proportion of accumulated dislocations and cross-slip occurring is smaller.

For the perfect cube, Figure 6c,d displays a few slip lines on the surface due to the non-ideal aligned compression, just like those which were observed for the aligned cube. The cubic shape of the particle is also retained. This implies that the difference in alignment can cause changes in the deformation process, especially in ductility. The variation in plastic strains arises due to the combination of size effect and alignment, which is positively related to the corresponding number of activated slip systems. In the Figure 6e, the double cube is composed of two side-by-side nanocubes and one thin γ phase slice connecting them. Two slip systems were activated on the surface of nanocubes, as shown in Figure 6f. The increase in section area ensures a better alignment of in-situ compression. As a result, the particle undergoes a significant change in dimensions and numerous slip planes appear on the surface of the dislocation-free Ni_3_Al nanocubes, which is consistent with Shan et al.’s [21] result. They found that moving dislocations inside the nanocrystals have a much higher probability of annihilating at the free surface rather than reacting with each other at the intersecting slip planes to form a dislocation lock. As no effective barriers are present to impede dislocation motion, the nucleated dislocations run out of the nanocubes with a high velocity. Similarly, it seems that the γ–γ’ interface cannot prevent the dislocation from gliding throughout the entire double cube (see Figure 6f).

### 3.3. Crack Initiation Mechanism for Ni_3_Al Nanocubes

Although extrinsic cubic shapes are not supposed to affect the yield strength, they may change the slip line distribution and hence influence compressive plasticity. For a given micropillar, according to Greer et al.’s deduction, the stress required to propagate a shear band to fracture is [22]:

(2) $$\sigma_{fracture}=2^{3/4}\sqrt{\frac{\Gamma E}{\beta d}}$$

where Γ is the shear band energy density, *E* denotes Young’s modulus, *d* is the diameter of the pillar and β represents the aspect ratio (H/W), leading to $\sigma_{fracture}\propto H^{-1/2}$.

The above criterion is usually applicable to the classical approach in brittle failure analysis. For the Ni_3_Al nanocubes, the shear band energy density can be roughly replaced by the stacking fault energy. On the other hand, the typical stress size scaling trend for fcc nanocrystals can be simplified as:

(3) $$\sigma_{flow}\propto W^{-0.6}$$

The stress drop between the yield strength and fracture strength is a result of the feedback loop that controls the displacement rate. The feedback control recognizes that the present displacement is too far relative to the programmed value at that time. For the pillars, each slip event is followed by a stress drop [23], while in the nanocubes, all the slip events were accumulated to one apparent stress drop. Maaβ et al. [23] noted that the strain-jump magnitude increases with decreasing pillar size. They considered that the strain-jump is a signature of a dislocation avalanche. Compared with the FIB-imaged Ni_3_Al pillars, the dislocations in the as-extracted nanocubes are difficult to intersect with one another due to the geometrical constraints and are more likely to annihilate at the cube surface, which corresponds to only one strain-jump, as shown in Figure 3.

**Figure 7:**
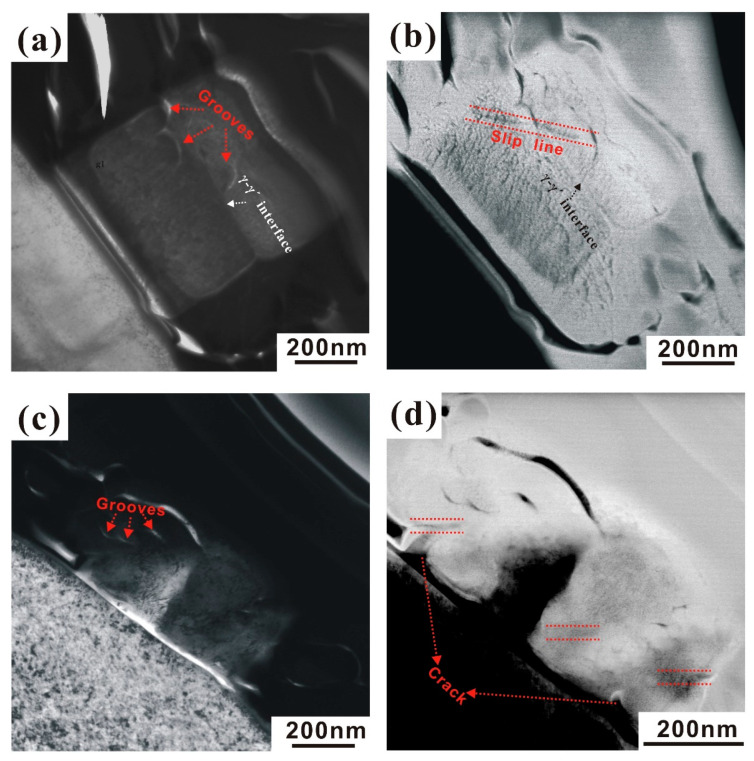
Crack initiation of various shaped Ni_3_Al nanocubes and formation of grooves at γ–γ’ interfaces. (**a**,**b**) Aligned cube; (**c**,**d**) double cube.

Furthermore, Figure 7 compares crack initiation between the aligned cube and the double cube. In Figure 7a, several grooves appear at the γ–γ’ interface. The grooves and ledges usually form during heat treatment and creep [24]. Parsa et al. [13] found that grooves and ledges result from local dissolution events which are triggered by dislocations at γ–γ’ interfaces. They established a thermodynamic model of how dislocation stress fields change the local chemical potentials and drive diffusional fluxes, resulting in the formation of a groove. Similar to Figure 5b, Figure 7b shows that a slip line runs through the γ phase and intersects with the groove, which is beneficial for the preferred initiation of a crack at this position and subsequent crack propagation along the slip plane. In the double cube, the grooves also appear at the edge of the cube (see Figure 7c). It can be seen in Figure 7d that multiple slip systems were activated within the cubes. The crack initiated at the corners of cubes, and seems to be related to the presence of interfacial grooves. For homogenous deformed nanocubes, crack initiation is promoted by shape-imperfect positions based on the intersections between slip systems. Once nucleated, the crack further propagates along one or more slip planes.

## 4. Conclusions

In the present work, we study the deformation behavior of dislocation-free Ni_3_Al nanocubes during in-situ compression. This result emphasizes the effect of cubic size and aspect ratio on the yield strength and nanocube plasticity, respectively. Together with the crack initiation mechanism of Ni_3_Al nanocubes, the following conclusions can be drawn:

(1) The yield strength of dislocation-free L1_2_ nanocubes with sizes larger than 100 nm exhibits a clear size dependence. When the isolated Ni_3_Al nanocubes are below 300 nm, the size dependence significantly increases. Compared with the strength value of bulk Ni3Al single crystals, the normalized deformation strength of nanocubes is two orders of magnitude higher, which proves that there is a size effect.
(2) Unlike the yield strength, the plastic strain mostly depends on the alignment and the shape of cubes. For aligned and perfect cubes, the deformed aligned nanocubes display only a few slip lines and keep their macroscopic shape. The other three as-extracted nanocubes were homogeneously compressed into flat square-shaped discs formed by multiple slip dislocation-mediated plasticity. This shape change is finally accompanied by a rapidly occurring post-yield catastrophic failure.
(3) For the aligned cube, the crack initiated at the intersection between the slip plane and grooves and subsequent crack propagation occurred along the slip plane. For the double cube, the crack initiation gives priority to the shape-imperfect position, which further propagates along one or more slip planes.
